# A Study on CKD Progression and Health Disparities Using System Dynamics Modeling

**DOI:** 10.3390/healthcare10091628

**Published:** 2022-08-26

**Authors:** Ahmeed Yinusa, Misagh Faezipour, Miad Faezipour

**Affiliations:** 1 Computational and Data Science Program, Middle Tennessee State University, Murfreesboro, TN 37132, USA; 2 Department of Engineering Technology, Middle Tennessee State University, Murfreesboro, TN 37132, USA; 3 School of Engineering Technology, Electrical and Computer Engineering Technology, Purdue University, West Lafayette, IN 47907, USA

**Keywords:** chronic kidney disease, healthcare disparities, minority populations, healthcare intervention, system dynamics modeling

## Abstract

Chronic kidney disease (CKD) is one of the most prevalent national health problems in the United States. According to the Center for Disease Control and Prevention (CDC), as of 2019, 37 million of the US’s adult population have been estimated to have CKD. In this respect, health disparities are major national concerns regarding the treatments for patients with CKD nationwide. The disparities observed in the healthcare interventions for patients with this disease usually indicate some significant healthcare gaps in the national public health system. However, there is a need for immediate intervention to improve the present healthcare conditions of minorities experiencing CKD nationwide. In this research, the application of system dynamics modeling is proposed to model the CKD progression and health disparities. This process is based on the health interventions administered to minorities experiencing CKD. The graphical results from the model show that there are relationships among the dynamic factors influencing the incidence and prevalence of CKD. Hence, healthcare disparities are inherent challenges in the treatment and management of this disease.

## 1. Introduction

### 1.1. Background

Chronic kidney disease (CKD) is a prevalent global and national health concern, affecting people from all backgrounds, ethnicities/races, and genders in the United States. It is an irreversible and progressive disease that results from the kidneys’ inability to filter blood and adequately eliminate wastes and poisons.

There are five stages of kidney disease. The eGFR (estimated Glomerular Filtration Rate) is generally used to determine the stage of the disease. As the kidney stage progresses, the eGFR number decreases [1].

CKD starts with undiagnosed kidney damage and develops into complete kidney failure, end-stage renal disease (ESRD). At this stage, the individual involved will require a total kidney transplant or replacement for survival [2]. Often, CKD comes with a higher risk of comorbidities, such as hypertension, diabetes, cardiovascular disease, and complications with financial and physical challenges on patients and the concerned communities, usually resulting in clinical cost implications and sometimes death [3]. The intensity in its prevalence differs based on several factors because it affects patients at a different rate. As of 2019, approximately 37 million adults in the United States (about 15% of the population) have CKD [4]. This chronic disease represents the ninth leading cause of death nationally [4]. Because of the financial and economic commitment involved in the procedures and treatments at every stage of the disease, minority populations in the United States, including Black or African Americans, American Indians or Alaska Native, Native Hawaiian or Pacific Islanders, Asians, and Hispanic Americans, usually experience disparities in healthcare intervention toward CKD. Its high mortality rate is rampant among these populations. Complex interacting nontraditional risk factors, such as physical environmental, social–economic, and genetic factors, contribute to the racial/ethnic CKD health disparities among the minority populations in the United States [5]. Moreover, despite the vast cost (around USD 81.8 billion in 2018) of the budget involved for CKD Medicare beneficiaries, CKD health disparities still exist. The disproportionate burden of CKD incidence and prevalence is a global concern; hence, it creates emotional, physical, financial, and mental stress for the patients involved. Healthcare intervention should be unbiased to improve the health of CKD patients. Generally, healthcare disparities are apparent in the United States because it is home to many different individuals with various racial or ethnic backgrounds. Subsequently, these populations get access to the healthcare system differently. In several chronic diseases, particularly CKD, the health disparities have increased the disease’s progression.

The study dives deep into the root causes of CKD and its different challenges present in the minority-populated communities using system dynamics to model, simulate, and visualize the possible healthcare disparities involved. In addition, the system dynamics modeling tools are utilized to analyze the causes and impacts of the various CKD risk factors involved. A simulation of the system dynamics model design is performed in different run tests and scenarios. This research employs a systems engineering application to address the complexities of CKD incidence, prevalence, and the health disparities involved in the treatment interventions, specifically in the state of Tennessee.

Furthermore, mathematical equations of the variables that represent the non-linear (dynamic) relationships and the nature of the CKD risk factors, and their connections are developed using system dynamics modeling.

### 1.2. Literature Review of CKD Health Disparities

The health of different cultures and societies can vary dramatically, and specific populations may have higher disease rates than others. As discussed earlier, inequalities in health or medical issues can substantially impact a community’s public health. Moreover, CKD health disparities represent the disproportionate outcome of the affected populations’ treatment, particularly the minority communities. At the same time, CKD is the outcome of a complex interaction of genetic and environmental factors, which adversely affect the lifestyle and wellbeing of humans.

CKD is prevalent in its causes and effects on the minority populations in the US. Therefore, this section of the paper reviews the literature of the previous studies on the health disparities involved in the healthcare intervention for CKD patients in these minority populations. Because various interacting factors (genetic, environmental, socioeconomic, etc.) are associated with the cause and incidence of CKD, it is necessary to delve deeply into the root causes of the disease.

In the case of CKD care, primary care providers have considerable roles to play in educating individuals to identify the causes and symptoms of this disease early on. At the same time, creating awareness of known racial, economic, and ethnic disparities in CKD care can improve the healthcare intervention for the most vulnerable populations. A disproportionate number of Americans, mainly African Americans, die from CKD [6]. The issues of CKD health disparities have been discussed extensively in the related research work [7,8,9,10,11,12,13,14,15,16,17]. Moreover, individuals who might otherwise be denied medical care due to their socioeconomic situation, lack of insurance, or underinsurance can now receive it. Those from ethnic minorities who are poor or ignorant about healthcare are the majority who rely on safety-net services.

Jenna et al. suggested that CKD results from complex genetic (age, treatment, developmental programming, etc.) and environmental factors that reflect the balanced interaction of nature and nurture [18]. Their research emphasized the strong effect of the social determinant of health (socioeconomic status, psychosocial factors, healthcare access, and neighborhood) as an essential environmental component, particularly for Black and other minority populations. Their study also emphasized that patients have numerous social aspects that must be understood by primary care providers, nephrologists, and other CKD medical experts who care for them and how these concerns may interfere with effective disease treatment. Keith et al., in their research, explained that several studies had indicated disparities in CKD incidence, prevalence, and management across various minority populations [19]. However, many aspects of this chronic disease, including the existing inequities, remain unknown despite this progress. Besides modifiable risk factors (socioeconomic status, environmental factors, culture, ethnicity, and access to healthcare), other risk factors, such as biological factors associated with CKD, are still unclear. Susanne et al. discussed that several socioeconomic factors, such as poverty and unemployment, have been related to kidney disease progression and death, leading to ESRD [20]. Furthermore, Winfred et al. explained that to uncover and deconstruct the structural drivers of systematic racism in the healthcare profession, efforts have been made since spring 2020. Based on race, disparities in the predicted total renal filtrate flow rate have spawned several controversies in recent years [21].

To transparently address the incidence, prevalence, and healthcare in CKD, it is clinically and socially necessary to understand the basics and the sources of these health inequities by independently considering the patients’ lifestyle and the social determinants of health. We also need to address the bias on the side of the physicians and the healthcare system. To this end, this research study addresses the health disparities surrounding CKD by factoring in the vital variables involved in its occurrence and how these variables influence the disparities therein.

### 1.3. Systems Engineering and System Dynamics

Systems engineering and system dynamics have been applied to many areas within healthcare and sustainability [22,23,24,25,26,27,28,29,30,31,32,33,34,35], as well as areas within smart healthcare [36,37,38,39].

Generally, factors associated with the incidence of CKD are interrelated. These factors create dynamic and complex challenges that cannot be addressed at the surface using a conventional approach. Because these factors are interactive, analyzing them linearly to provide a possible panacea to CKD’s causes and health disparities would be tasking. For this reason, to proffer equal, fair, and considerable healthcare intervention for CKD patients across the board nationwide, it is necessary to carefully consider the root causes of CKD health disparities rather than addressing the after effect involved. Furthermore, to address the overburdened healthcare disparities associated with CKD in Tennessee, a systems engineering concept called the system dynamics modeling approach is employed in this research.

System dynamics is a mathematical modeling technique that simulates, analyzes, and helps comprehend complex subjects, problems, and systems. It adopts causal loops (cause and effect relationships), stocks, flows, time delays, and feedback concepts to understand the non-linear characteristics of dynamic systems. It employs a real-time mathematical technique to present the policy-oriented results of events. It also observes how the feedback mechanisms of complex systems create or cause events around us. This concept of modeling was developed originally in the mid-nineteenth century, in the 1950s, at MIT by Jay W. Forrester [40] to address issues in various fields ranging from engineering, education, economics, psychology, and biology to management. It is currently employed by both the public and private sectors for decision analyses and policy designs.

## 2. Methodology

This section explains the methodology, design, simulation software, data collection, and study analysis for addressing CKD health disparities using system dynamics modeling. Because health disparity is prevalent in the nation’s healthcare system, it is necessary to find the root causes. Because CKD is irreversible, a well-planned healthcare intervention is needed to curtail its progression. As health disparities are inherently associated with CKD nationwide, this study focuses on Tennessee’s CKD healthcare disparities. Despite the Medicare, Medicaid, and Medigap health insurance plans provided by both the federal government and Tennessee, these health disparities are still prevalent in the state.

The methodological approach adopted involves simulating various interactive factors involved in the CKD incidence, prevalence, and healthcare disparities. CKD has several risk factors associated with it, and these factors are complex and dynamic with interrelated characteristics. To understand the inequities in the care of patients with CKD, a dynamic modeling approach is appropriate. This study employs a systems engineering modeling concept called system dynamics modeling to simulate various instances associated with health disparities in CKD. This modeling concept creates graphs to illustrate the relationships that exist among the CKD factors. Hence, the results are analyzed based on the correlations to provide a proper CKD healthcare intervention for the minority communities in Tennessee. To do this, all the variables necessary in CKD care are utilized for transparency and policy decisions.

### 2.1. Causal Loop Model

The system dynamics modeling software used is Vensim PLE [41], which serves as the modeling environment. This software is employed for both causal loops, stock, and flow models. This section utilizes the system dynamics casual loop model to explain the complex interacting factors associated with CKD in the minority communities in Tennessee.

The causal loop model is a cause-and-effect tool used in system dynamics modeling to create interactive relationships between variables such as causes, events, and processes. It utilizes descriptive arrows to explain the balancing and reinforcing loops involved in events. This looping idea is essential to critically explain the causes and effects in complex situations that an ordinary linear approach cannot illustrate. A causal loop model is basically of two types: balancing loop (B) and reinforcing loop (R). A balancing loop is produced when a variable change (effect) is generated due to an opposite variable’s change (cause). While when a variable change (cause) in one direction results in more variable change (effect) in another direction, a reinforcing loop is generated. A positive sign (+) is assigned to the path of X to Y when a variable, Y (effect), moves in the same direction as a variable, X (cause). Additionally, when the variables move in opposite directions, a negative sign (−) is assigned to their path. The causal loop diagram approach is appropriate to illustrate the challenge with CKD care in Tennessee. As initially explained in the previous sections, there are factors associated with CKD incidence, prevalence, and management. CKD health disparities are better understood, considering the biological, environmental, and social factors involved.

In this model, to describe the minority communities for better understanding, a single system dynamics stock is used to represent these communities (Black/African Americans, Asian Americans, Hispanic Americans, American Indians/native Alaska Americans, and native Hawaiians/Pacific Islanders).

It is essential to have a clear grasp of how social determinants of health may affect kidney health. Many patients learn that they have chronic kidney condition after the disease has already advanced. The delayed diagnosis of kidney illness can be attributed to social determinants of health, such as inaccessibility to preventative care in one’s community or challenges in traveling to a physician’s office.

**Figure 1:**
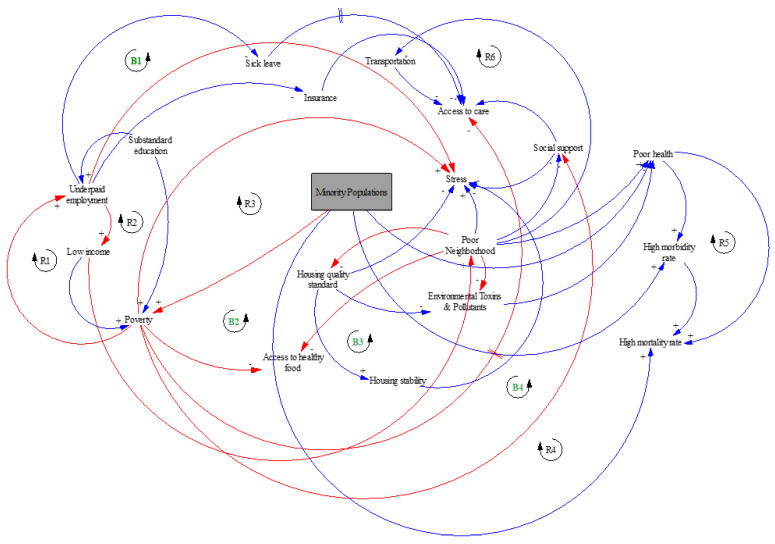
The Causal Loop Model for the Influence of Social Determinant of Health on the Minority Populations. This diagram shows how social determinant of health factors impacts the incidence and the prevalence of CKD in the minority populations, with the reinforcing (R) loops and balancing (B) loops displayed. The red colored arrows represent the disparities, and the blue arrows signify the usual and general influence of social factors on the health of the minorities.

Figure 1 is a causal loop diagram in system dynamics that provides illustration of all significant relationships. It shows the relationship between important factors in a visual way. Such diagrams display variables as text boxes and arrows as evidence of the causal connections between them. The variables (or factors) involved in the causal loop diagram in Figure 1 are the predominant social determinants of health factors influencing the incidence and progression of chronic kidney disease [8,9,18,20,42], which are major influencers on the risk factors associated with this chronic condition.

The causal loop model in Figure 1 exemplifies the impact of social factors on the wellbeing and healthcare interventions in minority communities. The arrows in red in the causal loop represent the disparities that influence the social determinants of the health of minority populations by identifying the root causes of the inequity involved in the incidence, prevalence, and healthcare intervention of CKD. Factors such as poverty, stress, underpaid employment, low income, inadequate access to healthy food, poor housing, environmental toxins, pollution, poor neighborhood, discrimination, less access to care, inadequate social support, and poor health insurance coverage limit CKD minority populations’ access to equal healthcare intervention nationwide. Blue arrows signify the usual and general influence of social factors on the health of the minorities. The green-colored balancing loops (B1, B2, B3, and B4) indicate the impacts of social factors on the health of minority populations when an increase in one of these social determining factors results in a decrease in the other. The black-colored reinforcing loops (R1, R2, R3, R4, R5, and R6) indicate the influence of the social factors in the same direction. In the model, loop R1 creates an influence by showing that underpaid employment leads to low income, resulting in poverty and, in turn, unemployment. In loop R2, substandard education results in underpaid employment. Furthermore, in loop R3, the underpaid employment and poverty in the minority populated areas usually lead to stress. For loop B1, an increase in underpaid employment gives access to less sick leave. Moreover, less sick leave does provide limited access to standard medical care. At the same time, unemployment results in low health, housing, and motor insurance. Loop B2 creates an influence on limited access to healthy food because of poverty and poor neighborhood. In loop B3, standard quality and stable housing usually reduce stress and environmental toxins and pollutants. Furthermore, in loops R4 and R5, the high mortality rate in the minority populations is due to a high morbidity rate and poor health. In loops B4 and R6, there is usually lower social support when there is poverty which at the same time provides limited access to care and poor transportation in these communities.

### 2.2. System Dynamics Modeling

The social determinants of health play a vital role in the incidence of CKD because it is highly connected with risk factors associated with this disease. The influence of these social factors can necessitate the earlier incidence of the risk factors. People with risk factors for chronic kidney disease, such as diabetes mellitus, high blood pressure, poor dietary patterns, and unhealthy lifestyle patterns, are disproportionately represented in low-income communities and neighborhoods.

To fully understand CKD’s root causes, impacts, and health disparities, it is crucial to view the relationships among the dynamic complex factors involved in its incidence and prevalence. System dynamics model can establish the relationships within these varying factors and shows the possible CKD healthcare disparities presented by the patients (populations), healthcare system, the primary care providers, and nephrologists. In this research, the system dynamics model displays vital graphs of the relationships and correlations with CKD health disparities. These graphs depict the implied interacting factors to establish possible healthcare disparities at every stage of CKD. The rate variables are on percentage rate units. All other stocks are kept at zero initial values except the population stock. Moreover, each rate variable is based on a percentage rate approximated to two decimal places to avoid model complications and variable overflow.

In this model, some variables are used as stocks, while others as flows. Stocks are quantities or variables that can increase or decrease in value. At the same time, the term “flow” refers to the ability of an element or a variable to change over time.

**Figure 2:**
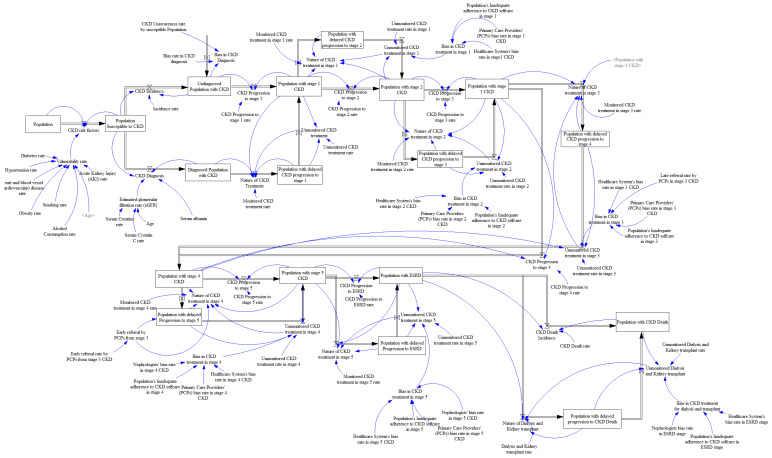
System Dynamics Modeling Representing CKD Incidence, Prevalence, and Health Disparities.

Figure 2 is a stock and flow model in system dynamics that represents how a system is understood structurally, illustrating the causal chains that lead to the behavior that is seen. It provides information about the rates of change in the system components (variables) and the values of the variables.

The system dynamics model employed in this research (Figure 2) involves possible factors necessitating the incidence, prevalence, and health disparities associated with CKD. The system dynamics model in Figure 2 considers the impacts of the social determinants of health on the risk factors with the biases existing at every stage of CKD to monitor the CKD progression.

## 3. Simulation Results and Graphical Interpretation

This section explains the relationships among the varying factors associated with CKD, resulting from the system dynamics model in the previous section. The CKD incidence occurs due to the prevalence of the risk factors present in the general population, specifically the minority populations susceptible to CKD. As noted in various studies [5,7,18,19,42,43,44,45], the bias in CKD treatment and management is assumed to be a cumulative disparity between patients, primary care providers, nephrologists, and healthcare systems in this research. In the model, the bias arises from the population’s inadequate adherence to CKD self-care and the primary care providers’, nephrologists’, and the healthcare system’s biases toward CKD. The nature of treatment in the model is based on the monitored treatment and the unmonitored treatment experienced by the patients. To test the presence of CKD in a patient, several tests are involved. The ones employed in the model are the estimated glomerular filtration rate test and the patient’s protein urine (Serum albumin) [46].

### 3.1. Graphical Interpretation

This section interprets the results generated by the graphs in the system dynamics model using the modeled variables: CKD risk factors, the population at every stage, the nature of the treatment, bias, and the progression of CKD to different stages. Each of these variables are plotted against each other to initiate the desired results of the study. The following graphical illustrations show and explain the variations of the different biases that can exist at every stage of CKD and how these bias impacts can result in the progression of this disease. In the figures below, the primary care providers’ bias rates, which are part of the bias influence in CKD treatment at every stage, are plotted against the CKD progression to subsequent stages. In the following graphs, the base simulation of the model is displayed. Afterward, the current simulation presents the change in the results when one or two CKD factors are altered. The current simulation is based on the change in diabetes rate (0.80 to 1.15 Percentage Rate) and hypertension rate (0.80 to 1.25 Percentage Rate). To have proper simulations and graphical results, the current population of Tennessee, which is 6,910,840, according to the 2020 census [47], is entered into the population stock in the model. The time duration for the model for the provision of the desired results is from 2010 to 2022. This duration is employed to give enough room for simulation comprehension because the incidence and prevalence of CKD take a long time to occur.

#### 3.1.1. CKD in Stage 1 and the Bias Involved

Healthcare disparities can occur at every stage of CKD, from its incidence to its prevalence. At the earlier stages of CKD, especially at stages 1 and 2, most patients are asymptomatic, that is, they do not know they have this chronic condition. Any slight form of bias from the patients themselves, in the form of nonadherence to CKD care, or unmonitored CKD treatment by the primary care providers, the nephrologist, or even from the healthcare system, can trigger the prevalence and the progression of kidney disease at these earlier stages to further stages. Moreover, it is crucial to examine the inequities that might arise at stages 1 and 2.

**Figure 3:**
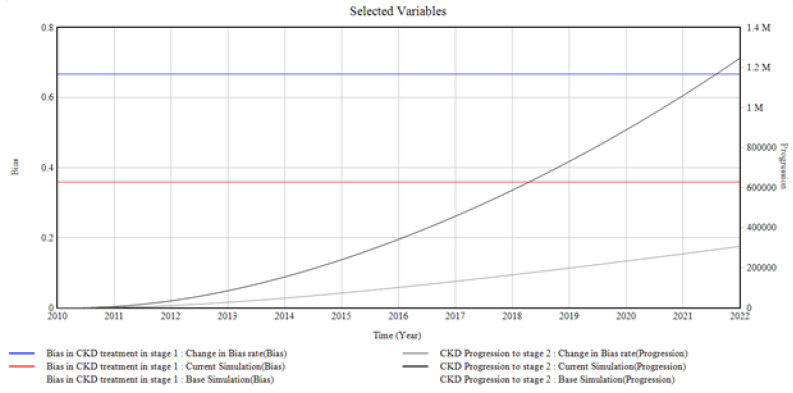
The Bias Rate in CKD in Stage 1 vs. CKD Progression to Stage 2 CKD.

From Figure 3, it is observed that the unmonitored CKD treatment in the model as a result of the bias in CKD treatment presented by the patients, the primary care providers, and the healthcare system enhances the progression of CKD to stage 2. The change observed in the graph occurred when the primary care provider’s bias rate, which is part of the bias in CKD treatment in stage 1, increased, resulting in the progression of CKD to stage 2. In this case, the primary care providers’ bias rate is changed from (0.8 to 1.3 Percentage Rate), giving rise to CKD progression in stage 2 to a value of (1.24 M Progression) in 2022 from 0 in 2010.

#### 3.1.2. CKD in Stage 2 and the Bias Involved

**Figure 4:**
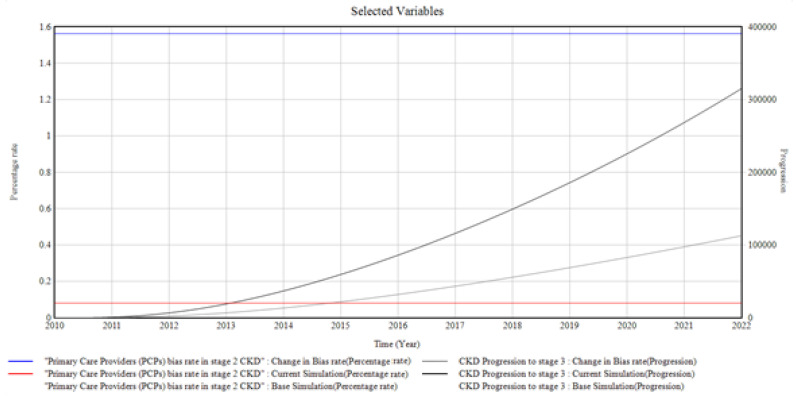
The bias in CKD treatment in stage 2 and the CKD progression to stage 3.

Figure 4 shows the trends and relationships among the factors influencing CKD stage 2 incidence and prevalence.

At stage 2, the overall bias experienced is also from the bias in CKD treatment, which is in the form of bias from the patients, the primary care providers, and the healthcare system. The primary care provider’s bias toward unmonitored CKD at stage 2 influences CKD development to stage 3. The graph shows a change in CKD progression to stage 3 when the bias in CKD treatment in stage 2 is increased correspondingly. In this case, the primary care providers’ bias rate is changed from (0.08 to 1.55 Percentage Rate), giving rise to CKD progression in stage 3 to a value of (310,000 Progression) in 2022 from 0 in 2010.

#### 3.1.3. CKD in Stage 3 and the Bias Involved

**Figure 5:**
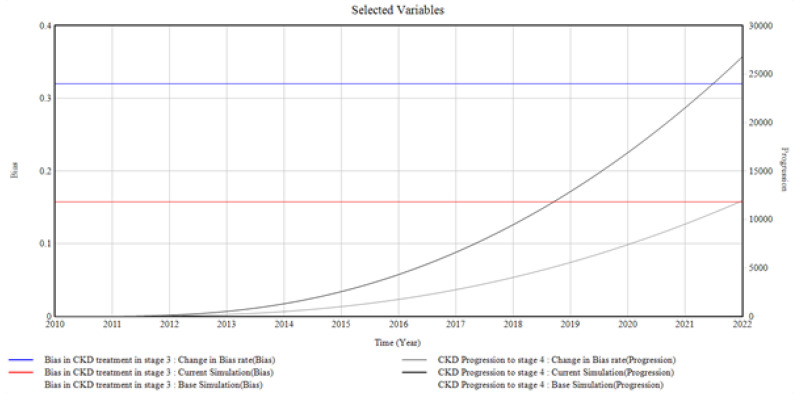
The Bias Rate in Stage 3 CKD vs. CKD Progression to Stage 4 CKD.

Figure 5 illustrates the trends and relationships among the factors influencing CKD stage 3 incidence and prevalence.

The primary care provider’s bias at CKD stage 3 in terms of unmonitored CKD treatment at this stage affects CKD progression to stage 4. The graph depicts a proportional increase in CKD progression to stage 4 when the bias in CKD treatment in stage 3 is changed. The bias rate, in this case, is increased from (0.16 to 1.32 Bias), giving rise to CKD progression in stage 4 to a value of (26, 5000 Progression) in 2022 from 0 in 2010.

#### 3.1.4. CKD in Stage 4 and the Bias Involved

**Figure 6:**
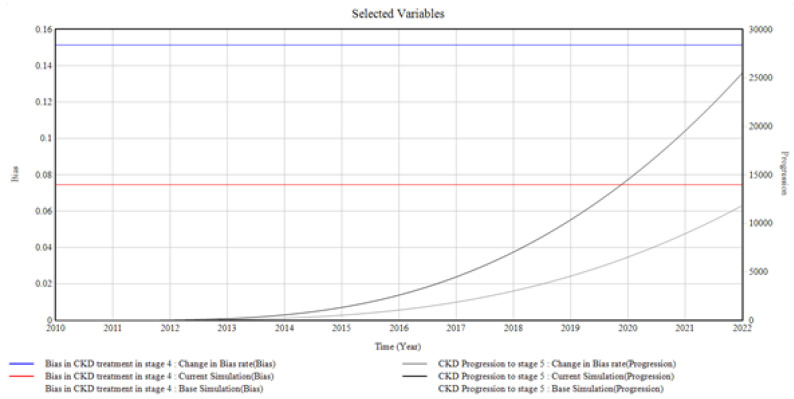
The Bias Rate in Stage 4 CKD vs. CKD Progression to Stage 5 CKD.

Figure 6 depicts the trends and relationships among the factors influencing CKD stage 4 incidence and prevalence.

The progression of CKD to stage 5 is influenced by the bias initiated by the primary care provider in CKD stage 4. The graph shows a corresponding increase in CKD progression to stage 5 when the bias in CKD treatment in stage 4 is altered. The bias rate is changed from (0.073 to 1.151 Bias), giving rise to CKD progression in stage 5 to a value of (25, 1000 Progression) in 2022 from 0 in 2010.

#### 3.1.5. CKD in Stage 5 and the Bias Involved

**Figure 7:**
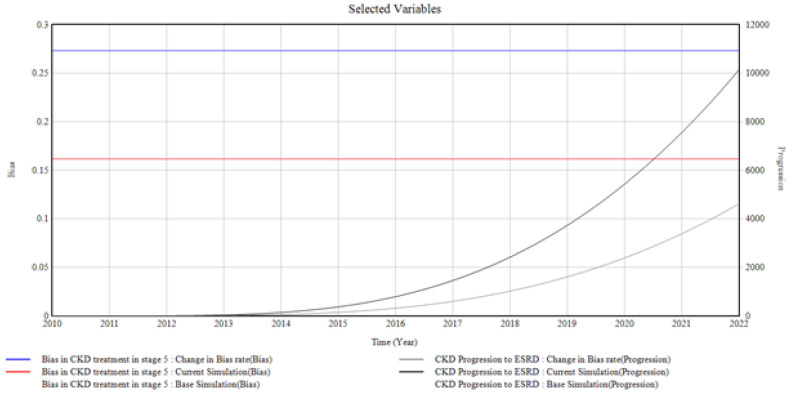
The Bias Rate in Stage 5 CKD vs. CKD Progression to ESRD.

Figure 7 shows the trends and relationships among the factors influencing CKD stage 5 incidence and prevalence.

The CKD unmonitored treatment experienced by the patients in stage 5 impacts CKD progression to ESRD. This arose as a result of the bias involved in stage 5. The graph shows an increase in CKD progression to ESRD when the bias in CKD treatment in stage 5 is changed. The bias rate is changed from (0.152 to 0.275 Bias), giving rise to CKD progression in ESRD to a value of (1000 Progression) in 2022 from 0 in 2010.

#### 3.1.6. ESRD and the Bias Involved

**Figure 8:**
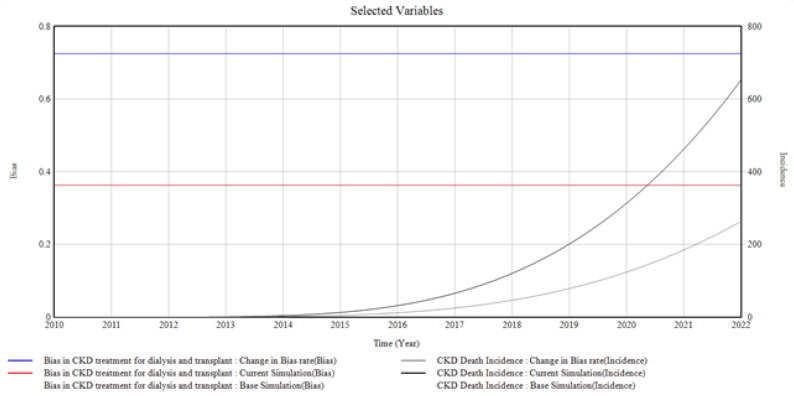
The Bias Rate in ESRD vs. CKD Death Incidence.

Figure 8 presents the trends and relationships that exist among the factors influencing ESRD incidence and prevalence.

The death incidence occurred because of the bias involved at the ESRD stage. The graph shows an increase in CKD death incidence when the bias in CKD for dialysis and transplant is changed. The bias rate is changed from (0.33 to 0.72 Bias), giving rise to CKD death incidence of a value of (650 Incidence) in 2022 from 0 in 2010.

Table 1 summarizes the CKD progression with the bias involved in each stage.

## 4. Discussion

Disparities in healthcare relate to the systematic inequalities that can be observed in the health status of various population groups. These disparities have significant consequences, both socially and economically, for individuals and society. Most patients from minority-populated areas, in one way or the other, usually experience some type of healthcare disparity. As previously mentioned, there are many agents involved in CKD healthcare treatment and management, and any form of healthcare disparities can advance the disease to ESRD, or even death.

ESRD attracts more attention globally, and hence, it takes a large part of the medical allocation to kidney treatment nationwide. Therefore, more concerns are raised at this stage of CKD because the kidney often requires dialysis or transplant. As previously mentioned, in Tennessee, the minority populations bear the most burdens that come with ERSD. For this reason, this research addresses the challenges associated with the bias present in the management of this disease.

According to the American Kidney Fund in Tennessee [48], 15,662 patients have been burdened with ESRD without dialysis treatment or kidney transplants in 2021. On the other hand, 11,495 people are on dialysis to stay alive, while 4167 have undergone kidney transplants.

From the models, the use of the causal loop model is to investigate the sources of the disparities. Figure 1 modeled the impacts of the social determinant of health within the minority communities to establish the root causes of the bias generally associated with CKD. Moreover, in addressing the influence of the bias in the medical management and treatment of CKD, the system dynamics model in Figure 2 created a simulation to analyze the health disparities from the patients, the medical experts, and the healthcare system. The current population of Tennessee, according to the 2020 census, served as the population stock in the model to produce the desired simulation results. Furthermore, each influencing factor associated with CKD was included in the model.

What differentiates this study from other studies regarding health disparities in CKD is the system dynamics modeling involved that can present mathematical and numerical results of bias in different stages. Conclusively, the model generated graphical results that showed the effect of the disparities in CKD healthcare interventions in the minority communities. The simulation result graphs depict the trends required to ascertain the existence of bias in CKD.

From the graphical illustrations and interpretations of the results, it is deduced that health disparities usually occur in the incidence of CKD and its progression. Often, these disparities are present inherently, whether intentionally or unknowingly, and their influences, most of the time, initiate the progression of CKD at every stage. Moreover, the CKD system dynamics simulation model can accommodate different bias rates presented by the patients, the primary care providers, the nephrologists, and the healthcare system by plotting various factors associated with bias in the incidence and progression of CKD. However, for the graphical illustrations in Figure 3, Figure 4, Figure 5, Figure 6, Figure 7 and Figure 8, the varying bias rates in CKD treatments at every stage are plotted against the CKD progression from stage 1 to CKD death incidence.

Furthermore, the varying values in Table 1 show that any slight input and influence of disparities at any stage of CKD can result in its progression. CKD incidence and progression can be delayed if appropriate measures are considered to observe any type of disparity that might hinder the healthcare intervention of this chronic condition nationwide, specifically in the minority-populated areas in each state. Patients with CKD, healthcare providers, and nephrologists can benefit from this model if they can apply actual values as inputs to the system. However, this may require the long-term data collection of disparities, which is beyond the scope of this paper. With the proper awareness of CKD treatment and management by primary care providers and nephrologists, healthcare disparities can be alleviated. Moreover, with the existence of cordial relationships between physicians and patients, disparities in healthcare can be curtailed. Patients need to be educated on the causes, incidence, and progression of CKD so that self-management will be feasible.

## 5. Conclusions

Unlike other chronic diseases, CKD comes with overwhelming physical, mental, and financial burdens. In its earlier stages, most people are asymptomatic, not knowing they have it. It is becoming a global concern with rising incidence and prevalence. Often, if CKD is not discovered and monitored earlier, it progresses gradually to kidney failure or ESRD, giving the patients two alternatives, either continuous dialysis or kidney transplant. Furthermore, CKD treatment and management involve loads of medical attention and expenses. The weight of all these conditions disproportionately affects the minority populations nationwide. Hence, it creates several burdens in these communities. As a result of the challenges involved in CKD incidence and prevalence, it is essential to address the treatment of this disease across the board. The healthcare intervention provided by the healthcare system should be unbiased to improve the health of CKD. Because the US is home to a diverse population with different racial and ethnic backgrounds, the minority populations’ CKD healthcare needs to be addressed as equitably as other populations. Therefore, specifically, this research focused on the minority communities in Tennessee. A systems engineering application was employed to address the healthcare interventions provided to these communities in Tennessee. The application was based on the system dynamics modeling to simulate various interrelated dynamic complex factors associated with CKD, beginning with the risk factors involved to the nature of the treatment rendered and the health disparities involved. Moreover, any form of CKD healthcare disparities can arise from the bias presented by either the CKD patients, the primary care providers, the nephrologists, or the healthcare system. Therefore, in this research, the various healthcare disparities that occur in CKD care are modeled with the risk factors and the nature of CKD treatments to observe the relationships between the bias in CKD treatment and its progression. The system dynamics model can also simulate different graphs that represent how the biases in CKD care from patients, nephrologists, primary care providers, and the healthcare system affect the progression of this disease.

The research showed that system dynamics could be applied to model various dynamics, interrelated factors in CKD incidence and prevalence to uncover the healthcare disparities associated with it. The development of the system dynamics model and the underlying simulation results would aid policymakers and decision-makers in the healthcare area to make more informed decisions regarding CKD health disparities.

## Figures and Tables

**Table 1:** The Variation of the CKD Progression with the Bias Involved in Each Stage.

CKD (Incidence/Progression)	Stage 1	Stage 2	Stage 3	Stage 4	Stage 5	ESRD
**Bias Rate (Bias)**	[1.24M], [0.8 to 1.33]	[310,000], [0.08 to 1.55]	[26,5000], [0.16 to 1.32]	[25,1000], [0.073 to 1.151]	[1000], [0.152 to 0.275]	[650], [0.33 to 0.72]

## Data Availability

No new data have been collected for this research. The simulations were performed using synthetic data with underlying assumptions in each scenario explained in the paper.

## Abbreviations

The following abbreviations are used in this manuscript:

CKD	Chronic Kidney Disease
ESRD	End-Stage Renal Disease
